# Identification and synthesis of end-of-life decision-making measures: a scoping review

**DOI:** 10.3389/fmed.2025.1540486

**Published:** 2025-07-31

**Authors:** Patricia Bucko, Miriam A. Novack, Emily H. Ho, Berivan Ece, Evan Burleigh, Molly A. Mather, Tatiana Karpouzian-Rogers, Elizabeth M. Dworak, Sarah Pila, Zahra Hosseinian, S. Duke Han, Peter A. Lichtenberg, Richard C. Gershon, Sandra Weintraub

**Affiliations:** ^1^Department of Medical Social Sciences, Northwestern University Feinberg School of Medicine, Chicago, IL, United States; ^2^Department of Psychology, Northwestern University, Evanston, IL, United States; ^3^Department of Psychiatry and Behavioral Sciences, Northwestern University Feinberg School of Medicine, Chicago, IL, United States; ^4^Mesulam Center for Cognitive Neurology and Alzheimer’s Disease, Northwestern University Feinberg School of Medicine, Chicago, IL, United States; ^5^Department of Psychology, University of Southern California, Los Angeles, CA, United States; ^6^Institute of Gerontology, Wayne State University, Detroit, MI, United States

**Keywords:** end-of-life, decision-making, aging, advance care planning, cognitive impairment

## Abstract

**Background:**

Cognitive impairment (CI) and related conditions are known to affect decision-making (DM), particularly in older adult populations. The intersection of CI and DM ability is crucial in end-of-life (EoL) care, where there is a confluence of heterogenous preferences and values often across different constituents (e.g., healthcare providers, family members, and proxies). Standardized questionnaires are necessary to characterize patients’ EoL decisions, preferences, and readiness, but the extent of available measures is widely unknown.

**Objective:**

This scoping review aims to summarize the current state of the literature regarding EoL decision-making measures. This effort supports the development of the Advancing Reliable Measurement in Cognitive Aging and Decision-making Ability (ARMCADA) research initiative, which seeks to develop a decision-making battery for use in older adults.

**Methods:**

Following the Arksey and O’Malley framework, we conducted a scoping review for multiple domains of DM in studies published between January 2018 and November 2023. Any paper that assessed or characterized DM in participants 45 years and older was extracted for the DM domain, population characteristics, and DM measures.

**Results:**

An initial search identified 16,286 articles, of which 705 were classified as assessing DM. Of those, 34 articles included measures of the EoL domain, and 28 unique measures were identified. The MacArthur Competence Assessment Tool for Treatment (MacCAT-T), the Decisional Conflict Scale (DCS), and the Decision Regret Scale (DRS) were the only assessments used more than once in the scoping review. Many studies assessed clinical populations, including those with CI/dementia (12%), cancer (24%), and chronic conditions (16%). Findings show that measures used at the end of life emphasize decisional preferences, efficacy, and conflict.

**Conclusion:**

Overall, this review highlights the lack of DM ability measures that can assess older adults’ capacity to make decisions regarding EoL-specific issues (e.g., life-sustaining treatment and pain management).

## 1 Introduction

Recent years have seen an increase in diagnoses of cognitive impairment (CI) and dementia as the world’s population ages (1). The intersection of aging and CI holds particular significance in end-of-life (EoL) care, given that cognitive decline is known to impact decision-making (DM) ability and that decisions affecting EoL are often irreversible and of profound consequence. EoL decision-making refers to the process of making choices about medical treatments and care options, as well as the overall approach to care for individuals nearing the end of their lives. These decisions often involve discussions about the use of life-sustaining measures, pain management, and the individual’s preferences for their final days (2). It is particularly important to assess an individual’s decision-making ability in the context of EoL given that advance care planning discussions are often delayed and issues around deciding about aggressive treatment (e.g., resuscitation planning), pain management, and general preferences near the end of one’s life often arise when a person has not engaged in discussing such topics previously (3). Other areas of the EoL domain require decisions about aspects of life such as awareness of future disability and perceived risk, whether one wants to extend their life as much as possible despite risks, decisions about living situations including assisted care facilities and caregivers, and the creation and initiation of plans to meet goals (4).

Despite the importance of this topic, there is a lack of consensus on the best way to assess DM ability in an end-of-life context. The topic is further complicated by the fact that critically ill patients often also have some degree of decisional impairment (5). Because of this challenge, EoL decision-making is sometimes diverted from the patient (5, 6) to a surrogate, despite the fact that surrogates may poorly represent an individual’s preferences or choices (6). In a systematic review conducted by Sellars et al. (7), it was noted that carers often experience uncertainty in making EoL decisions and adhering to advance care planning (ACP) preferences on behalf of persons with dementia. Indeed, we know little about how to manage the inclusion of these surrogates when impairment is apparent. Yet, systematic reviews of how persons with limited decision-making capacity are meaningfully involved in ACP interventions are rare (8).

It is important to distinguish between decision-making capacity and decision-making ability, as these terms are often used interchangeably. Decision-making capacity has a distinct legal and clinical definition that refers to an individual’s ability to make informed choices, typically assessed via a specific framework that includes the ability to understand relevant information, appreciate the situation and its consequences, reason about options, and communicate a choice. In contrast, decision-making ability is a less restrictive construct that reflects a person’s general means to make decisions in alignment with their values and goals, without significant lapses in judgment. It is not a legal threshold but rather a clinical and ethical consideration that evaluates how well someone can navigate complex choices, particularly in critical and emotionally taxing contexts, such as end-of-life care. While this scoping review aims to focus on decision-making ability measures, decisional preferences, or an individual’s values, beliefs, and priorities that guide how they make choices, is something that must be considered as they may be greatly impacted by decision-making ability.

Within the context of EoL decisions, it is important to distinguish between preferences and the ability to make decisions. A significant portion of EoL research focuses on conflict in decisions between multiple options or preferences, including how they want their pain to be managed, where they want to die, whether they want life-sustaining treatment, ongoing care needs, etc. (9–11). Ultimately, these topics are indirectly associated with DM ability; decision preferences, conflict, and regret are often consequences of impaired DM rather than components of DM ability itself (12). Moreover, individuals with CI often experience diminished self-esteem and confidence in expressing their opinions due to their awareness of the disease and associated memory loss, which can further impact how they express their decisions (12). Family caregivers and other healthcare proxies face similar dilemmas when making decisions on behalf of cognitively impaired persons (11, 13), all of which highlight the need for assessments that acknowledge the nuances of this topic. For this reason, DM assessment is necessary to ensure that individuals and other persons involved in the EoL process understand their situation and the range of possible consequences of their decisions.

When assessing DM ability for EoL care, many other considerations must be taken into account. For example, it may be difficult to ensure that a patient’s decision is consistent with their long-term values and beliefs, particularly in clinical populations with dementia or psychiatric disorders that may affect an individual’s judgment (14). It is also important to ensure that a patient’s decisions are made voluntarily and without the influence of others who may try to take advantage of this vulnerable population for their own benefit (14). In the case of physician-assisted dying (PAD), patients must be able to understand their diagnosis and condition, as well as alternatives to suicide, the possible consequences of survival with injury, and the impacts of their decisions on survivors (14).

Taken together, researchers and clinicians face many challenges when looking to assess EoL decision-making. The aim of this study is to review existing measures that can gauge patients’ decisional ability about EoL choices. This review will provide a foundation for an EoL care decision model that is not influenced by physician predictions and/or surrogate inclinations (5). This scoping review on EoL DM is part of a larger multidomain review to support the Advancing Reliable Measurement in Cognitive Aging and Decision-making Ability (ARMCADA) study (15). ARMCADA aims to develop and validate a battery of measures that assess multiple DM domains in older adults. In addition to providing a state of the field, the results from the scoping review will help support the identification of common DM tools used to develop a comprehensive suite of DM measures for use across health and cognitively impaired older adults.

To accomplish this goal, we conducted a scoping review to summarize the current state of DM ability measures available. Importantly, the review took a broad approach by including any paper that tested participants aged 45 years and older to assess decision-making abilities in mid-life and track potential earlier changes in cognitive changes and neurodegeneration. This research addresses gaps in EoL measurement; namely, that DM measures tend to focus on preferences, and often do not contain assessments of ability to plan for ones’ end-of-life care. Furthermore, focusing on middle-aged groups and above recognizes that end-of-life (EoL) decision-making can occur early in life—particularly when individuals are aware of a potential future illness, such as neurodegenerative diseases identifiable through biomarkers or genetic testing (16). While it is important to acknowledge the rise of populations impacted by cognitive impairment, it is also important to recognize that cognitive impairment is not exclusive to neurodegenerative diseases in older adults, such as Dementia of the Alzheimer’s Type (DAT), but also affects other clinical populations, including those with chronic illnesses and conditions that may lead to early cognitive changes (17–19). Understanding the range and properties of measures that address EoL DM ability will be of use to healthcare providers and families and will better allow patients to be active participants in deciding what their needs are at the end of their life.

## 2 Methods

### 2.1 Protocol and registration

The EoL domain scoping review is a subset of a larger parent scoping review that was conducted to support the development and validation of a comprehensive battery of decision-making as part of the Advancing Reliable Measurement in Cognitive Aging and Decision-making Ability (ARMCADA) study. This review aimed to identify measures of DM across a variety of domains. Information regarding the protocol, research questions, and respective domain definitions can be found in a common protocol manuscript (15). Because this study focused on non-human subjects, institutional review board approval was not necessary and was classified as non-human subjects research at Northwestern University (STU00220334).

### 2.2 Search strategy and inclusion criteria

The scoping review employed the methodological framework developed by Arksey and O’Malley (20) and reported it in adherence with the Preferred Reporting Items for Systematic Reviews and Meta-Analyses Extension for Scoping Review (PRISMA-Scr) (21). Following the identification of research questions (15), an inclusion criterion and search strategy were developed.

A clinical librarian searched EMBASE (Elsevier), MEDLINE (Ovid), PsycINFO (EbscoHost), Cochrane Library (Wiley), Web of Science (Clarivate), and Scopus (Elsevier) for studies that met our criteria for each DM domain and were published from 1st January 2018 to 6th November 2023. By concentrating on literature published in the past 5 years at the time of the review, we aimed to identify the measures most commonly used in current research and practice, as well as to highlight emerging gaps that may not have been evident in earlier studies. This approach provides a more accurate understanding of the current landscape of EoL decision-making science and informs future directions for research and clinical settings.

Language limits were not applied as long as the manuscript was written in English or had an English translation. The search query contained general terms pertaining to DM ability in aging populations, which were identical across all domains. Example keywords included “decision making,” “choice behav*,” and “decisional capacity.” The search also included terms specific to the end-of-life domain, including “advanced care planning,” “hospice,” “terminal care,” “long-erm care,” “care plan*,” “end of life,” “advance care,” “elderly care,” and “mental capacity” [see (15) for a comprehensive search strategy including all decision-making keywords].

Inclusion criteria for the larger DM scoping review were consistent across all domains. The review focused on cohort studies, case–control studies, and randomized controlled trials that assessed DM in participants 45 years of age and older (see Supplementary Table S1 for details). In the process of developing the search criteria, we chose to exclude articles that included measures on *only* shared decision-making, decision aids, and perceptual/low-level decision-making [e.g., the random dot motion task (22)], as these topics were tangential to our definition of decision-making.

### 2.3 Selection of eligible studies

Our initial search strategy identified the studies for consideration. Further eligibility was then determined based on three stages of screening: (1) title and abstract screening; (2) full text review; and (3) full text extraction.

#### 2.3.1 Title and abstract screening

Eighteen reviewers, who had achieved a minimum of 85% reliability on a training set of 20 articles, conducted a title and abstract screening from 10th November 2023 to 8th December 2023, using the online review tool Covidence (23). Each text was screened independently by two reviewers who determined whether it met eligibility criteria and should proceed to full-text review. Reviewers agreed on 89.5% of papers (*n* = 14573). Papers for which both reviewers agreed on inclusion advanced to full-text review, and papers for which both reviewers agreed on exclusion were excluded from further review. In cases where the two reviewers disagreed (*n* = 1705), the text was further screened and discussed by two expert scientists who came to an agreement on whether the paper satisfied inclusion criteria.

#### 2.3.2 Full text review

A full-text review was conducted on 1656 papers across all DM domains. It took place from 8th December 2023 to 22nd December 2023 and was completed by 13 reviewers. During full-text review, the full paper (along with any Supplementary materials) was screened independently by two reviewers to determine further inclusion. The criteria (both reviewers must agree) were the same as title and abstract screening; any disagreements (*n* = 334) were consequently resolved by a third reviewer.

#### 2.3.3 Extraction

Relevant data from each paper was extracted via Qualtrics (58). For each paper, we extracted the following content: (1) definition of DM, (2) the DM domain(s) being assessed, (3) the populations tested (age range, sample size, and clinical characteristics), and (4) the names of the measures used. For each individual measure, we also extracted information about the measure, including the language of the assessment, administration characteristics (in-person, remote, and self-administered), required technology (computer, pen-and-paper, tablet, smartphone, and other), and psychometric properties. During the extraction phase, studies could be further excluded if they did not include an assessment that met our definition of DM ability (i.e., reward learning tasks and value choice tasks), that involved shared decision-making, when the papers were gray literature (dissertations, white papers, etc.), or if the language of the paper itself was not in English and a professionally translated copy was unavailable.

## 3 Results

**Figure 1:**
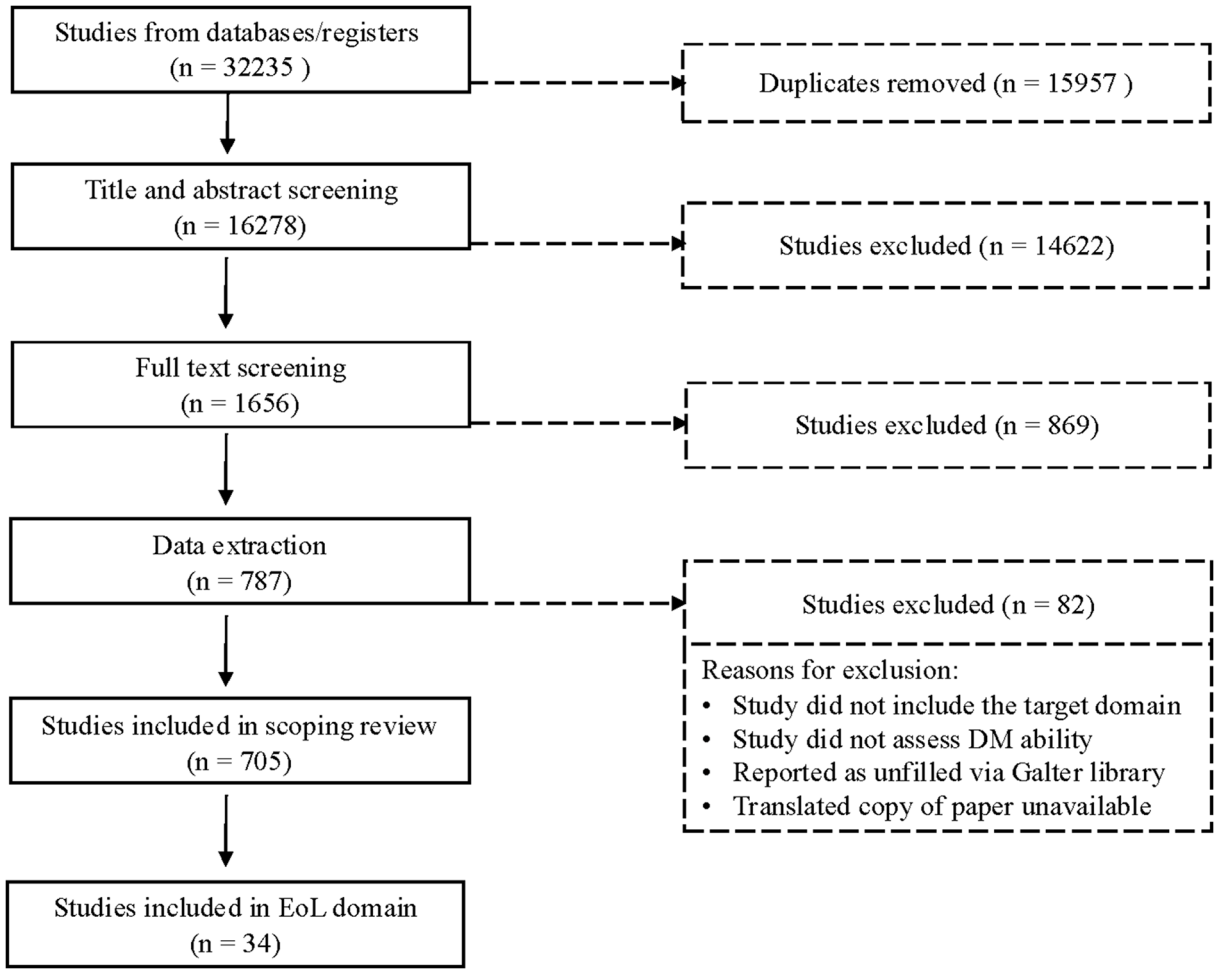
PRISMA flowchart of the article selection process.

In the original search, 32,235 articles were identified based on search criteria, 15,957 of which were duplicates and subsequently removed. The remainder (*n* = 16,278) were reviewed in Covidence. Stage 1 review (title and abstract screening) excluded 14,622 articles, while Stage 2 (full-text screening) excluded an additional 868. The remaining 787 articles proceeded to the extraction phase, during which an additional 82 articles were excluded; 705 articles were included in the final stage of extraction across all DM domains; 34 articles were identified as assessing EoL decision-making capabilities (see Figure 1). These 34 articles included 28 unique measures of decision-making (39 measure administrations total), which we review here.

### 3.1 Search results: measure-level

Of the 28 unique measures identified, the vast majority (*n* = 25, 89.3%) were used only by a single article in the past 5 years. The only three measures used by multiple articles were the MacArthur Competence Assessment Tool for Treatment [MacCAT-T; *n* = 7 articles; (24)], the Decisional Conflict Scale [DCS; *n* = 5 articles; (25)], and the Decision Regret Scale [DRS; *n* = 2 articles; (26)]. Of the 39 measure administrations in total, most instances of EoL measures (*n* = 31, 79.5%) were administered in person, with only 10.3% (*n* = 4) administered in a hybrid format (in person and remote) and 5.1% (*n* = 2) administered remotely. Seventy-two percent of measure administration (*n* = 28) were conducted as semi-structured interviews or questionnaires/vignettes led by an examiner, whereas only 25.6% (*n* = 10) took the format of self-report and one measure administration (2.6%), the EoL Care Preference Questionnaire (27), gave participants the option of being administered in an interview format and in a self-report format. We also identified that 59% (*n* = 23) of measures were in the form of a standard questionnaire or questionnaire and interview, 30.8% (*n* = 12) were vignettes or vignettes and interview, and 10.3% (*n* = 4) were exclusively an interview. Overall, the primary format of end-of-life DM measures found in the review was in-person examiner-administered semi-structured interview questions. Of the 28 unique measures identified in this review, only 21.4% (*n* = 6) of the measures assessed decision-making ability (Table 1) and the vast majority of the measures identified in this review focused on decision-making preferences (*n* = 18, 64.2%; Table 2) and decision-making outcomes (*n* = 4, 14.3%). See Supplementary Table S2 for complete extraction information listed by measure.

**Table 1:** Measures of decision-making ability and outcomes.

Measure name	Clinical group(s)	Administration details	Reporter	Decision construct	Psychometric information
MacArthur competence assessment tool for treatment (MacCAT-T; n = 7)^1,2,3,4,5,6,7^	Nursing home residents, cancer, Alzheimer’s disease, amyotrophic lateral sclerosis, dementia	In-person; semi-structured interview with vignettes; examiner-administered	Patient, caregiver, next-of-kin	Decision-making ability	*Dementia*: internal consistency (ω = 0.93)^5^
Decisional conflict scale (DCS; *n* = 5) ^8,9,10,11, 12^	Palliative care (cancer), dementia, chronic illness, renal disease	Hybrid; questionnaire or semi-structured interview; self-administered or examiner-administered	Patient, family member, caregiver	Decision-making outcomes	*Dementia*: internal consistency (*α* = 0.96)^8^ *Palliative care (cancer)*: Total Internal consistency (*α* = 0.85); Subscales – Uncertainty (alpha = 0.51), Informed (α = 0.83), Values Clarity (α = 0.93), Support (α = 0.42), Effective Decision (α = 0.47)^9^ *Dementia*: Internal consistency (α = 0.80–0.93)^10^ *Chronic illness (low-literacy version)*: Internal consistency (α ≥ 0.72), fair construct validity (most correlations ≥ 0.40, except Support subscale)^12^
Decision regret scale (DRS; *n* = 2) ^11,13^	Renal disease, prostate cancer	Questionnaire; self-administered	Patient	Decision-making outcomes	Renal disease: internal consistency (α = 0.81–0.92)^11^
Sure of myself, understand information, risk/benefit ratio, encouragement test (SURE Test)^14^	Intensive care unit, telemetry, medical, or surgical ward patients	In-person; questionnaire; examiner-administered	Patient	Decision-making outcomes	Not reported
View of Relocation Scale (VRS)^15^	Long-term care residents	In-person; semi-structured interview; Examiner-administered	Patient	Decision-making outcomes	Internal consistency: Subscales Perceived Control (KR-20 = 0.82), Perceived Need (KR-20 = 0.80)
Assessment of capacity to consent to treatment (ACCT)^16^	Unspecified mental health illnesses	In-person; Semi-structured interview with vignette; Examiner-administered	Patient	Decision-making ability	Not reported
MacArthur competency assessment tool for clinical research (MacCAT-CR)^17^	Cancer	In-person; semi-structured interview with vignette; Examiner-administered	Patient	Decision-making ability	Not reported
Willingness to accept life-sustaining treatment (WALT)^18^	None	In-person; semi-structured interview with vignette; examiner-administered	Patient, surrogate	Decision-making ability and preference	Not reported
Standard gamble assessment (SG)^19^	Primary intracerebral hemorrhage	In-person; Semi-structured interview with vignette; Examiner-administered	Patient, caregiver	Decision-making ability	Not reported
Advance care planning – capacity assessment vignettes method (ACP–CAV)^1^	Nursing home residents	In-person; semi-structured interview with vignettes; examiner-administered	Patient, next-of-kin	Decision-making ability	Not reported

^1^Kiriaev et al. (6); ^2^ Kolva et al. (30); ^3^ Kolva et al. (31); ^4^ Occhiogrosso et al. (32); ^5^ Poth et al. (1); ^6^ Santos et al. (33); ^7^ Spataro and La Bella (34); ^8^ Huang et al. (35); ^9^ Rego et al. (9); ^10^ Song et al. (8); ^11^ Tan et al. (36); ^12^ You et al. (37); ^13^ Wilding et al. (38); ^14^ Nair and Kohen (39); ^15^ Davison et al. (40); ^16^ Kotzé et al. (41); ^17^ Goswami et al. (42), ^18^ Batteux et al. (43); ^19^ Slaughter et al. (44).

**Table 2:** Measures of decision-making preference, consistency, and advance care planning (ACP).

Measure name	Clinical group(s)	Administration details	Reporter	Decision construct	Psychometric information
End-of-life care preference questionnaire^1^	None	In-person; questionnaire or semi-structured interview; self-administered or examiner-administered	Patient	Decision-making preference	Inter-rater reliability (0.91–0.98); test–retest reliability (0.94); Content validity (0.97)
End-of-life care survey^2^	Unspecified cancer	Remote; questionnaire; Self-administered	Patient, support person	Decision-making preference	Not reported
Problem-solving decision-making scale (portuguese version)^3^	None	In-person; semi-structured interview with vignettes; examiner-administered	Patient	Decision-making preference	Not reported
Advance care planning engagement survey – traditional Chinese (ACP-TC)^4^	None	In-person; vignette-based; administrator not reported	Patient	Decision-making preference and ACP	Internal consistency (α = 0.97); Test–retest reliability (r = 0.79–0.86)
Advance care planning questionnaire^5^	None	In-person; semi-structured interview or questionnaire; examiner-administered	Patient, accompanying person	Decision-making preference and ACP	Not reported
Advance care planning interview/questionnaire (ACP)^6^	Nursing home residents	In-person; semi-structured interview or questionnaire; examiner-administered	Patient, healthcare professionals	Decision-making preference and ACP	Not reported
AIP-LTC decision-making measure^7^	None	Format and administration not reported; questionnaire	Patient	Decision-making preference	Not reported
Confidence in surrogate’s ability to make healthcare decisions consistent with the older adult’s wishes^8^	None	In-person; semi-structured interview; examiner-administered	Patient, surrogate	Decision-making preference and consistency	Not reported
Decisional balance, attitudes, and practice behaviors of advance care planning (DAP-ACP)^9^	Cancer	In-person; questionnaire; self-administered	Patient	Decision-making preference and ACP	Internal consistency (α = 0.76–0.93)
End-of-life care decision scale^10^	Cognitive impairment	In-person; questionnaire; examiner-administered	Patient, family caregiver	Decision-making preference	Not reported
Life-support preferences questionnaire (LSPQ)^11^	Unspecified medical condition	In-person; semi-structured interview; questionnaire; examiner-administered	Patient, surrogate (family or friend)	Decision-making preference	Not reported
Patient expectation for participation in medical decision-making scale (PEPMDS)^12^	Colorectal, breast, or lung cancer	In-person; semi-structured interview; questionnaire; examiner-administered	Patient	Decision-making preference	Internal consistency (α = 0.89)
Patients’ perspectives on dialysis treatment decisions and end-of-life care survey (modified)^13^	Kidney disease	Hybrid; questionnaire; self-administered	Patient	Decision-making preference and ability	Not reported
Advance care planning acceptance questionnaire (ACP acceptance)^14^	Chronic condition(s)	In-person; Questionnaire; Self-administered	Patient	Decision-making preference and ACP	Not reported
Elderly death attitude scale^14^	Chronic condition(s)	In-person; questionnaire; self-administered	Patient	Decision-making preference	Not reported
Healthcare-related values and end-of-life preferences^15^	Unspecified mental health illnesses	In-person; semi-structured interview; examiner-administered	Patient	Decision-making preference	Not reported
Goals of care^16^	Dementia	Hybrid; semi-structured interview with vignette; examiner-administered	Patient, family caregiver	Decision-making preference	Not reported
Underspecified questions about organ donation for a relative^17^	None	Hybrid; questionnaire; administrator not reported	N/A	Decision-making preference	Not reported

^1^Ho et al. (27); ^2^Waller et al. (45); ^3^Gregório et al. (46); ^4^ Wei et al. (47); ^5^ Lim et al. (48); ^6^ Jin et al. (49); ^7^ Lindquist et al. (50); ^8^ Bravo et al. (51); ^9^ Chen et al. (52); ^10^ Huang et al. (53); ^11^ Ke et al. (54); ^12^Xiao et al. (55); ^13^ Saeed et al. (56); ^14^ Yang et al. (57); ^15^ Kotzé et al. (41); ^16^ Song et al. (8); ^17^ Liu et al. (59).

### 3.2 Search results: most frequently used EoL measures and constructs

1. *MacArthur Competence Assessment Tool for Treatment (MacCAT-T).* The MacCAT-T (24) was the most frequently used decision-making tool in the EoL domain. This tool, administered in a semi-structured interview format, measures four domains of competence: understanding relevant information that pertains to a condition and subsequent treatment, reasoning potential risk and benefits of choices regarding treatment, appreciating the nature of the situation and potential consequences of choices, and expressing a choice.
2. *Decisional Conflict Scale (DCS).* The DCS (25) is a 16-item tool that gauges the degree of uncertainty or difficulty an individual feels when making a decision, typically in healthcare settings. Along with uncertainty in making a decision, the DCS also measures modifiable factors related to decision-making, such as whether one feels informed enough to decide and has clear values and support guiding that decision. In the current review, the scale was used five times in an end-of-life context.
3. *Decision Regret Scale (DRS).* The DRS (26) is a 5-item tool designed to measure the extent of regret an individual feels after making a decision. It assesses how much an individual would have preferred to make a different decision and how much dissatisfaction they feel with the outcome of their decision. The DRS was used a total of two times in the end-of-life domain of this current scoping review.
4. *Measures of goal setting and preferences.* There was a total of 10 measures that addressed end-of-life goal setting and preferences. While there was variation in what these measures focused on, all of them touched on end-of-life preferences or end-of-life goal setting. Many of these measures brought up topics similar to those brought up in Advance Care Planning Assessments; however, they did not bring up advance care planning specifically. These topics included patient’s preferences regarding continuation of care (e.g., life-sustaining treatment versus pain alleviation), preferred surrogate decision-makers, and EoL values (e.g., valued activities at the end of one’s life).
5. Var*iations of Advance Care Planning (ACP) Assessments (Questionnaires and Interviews).* The scoping review did not identify a universal assessment of advance care planning; however, six studies included questionnaires that touched on common topics of advance care planning including but not limited to taking medical risks to lengthen ones’ life, quality of life preferences, proxy decision-maker preferences, concerns, and death preferences.

### 3.3 Search results: article-level

While our review selection criteria required that studies include participants who are at least 45 years old, we found that 100% of the articles (*n* = 34) that assessed end-of-life tested participants between the ages of 65 and 84, with the majority (61.8%; *n* = 21) including participants over the age of 85 years as well. Of the 34 articles, sample sizes ranged from just 11 participants to 17,193, with a median of 105 (Q1 = 51.5; Q3 = 297; IQR = 245.5). Forty-five percent (44.1%; *n* = 15) of articles reported that their measures were administered in only English, 14.7% (*n* = 5) articles reported that their measures were conducted in English and another language, and the remaining 41.2% (*n* = 14) reported that their measures were administered in a non-English language (Mandarin, Taiwanese, Portuguese, Italian, Malay, Korean, Japanese, French, and German). As expected, many of the articles included clinical samples, such as patients with cognitive impairment/dementia (*n* = 5, 14.7%), those with some type of cancer (*n* = 8, 23.5%), those with psychiatric disorders (*n* = 1, 2.9%), and those with another type of chronic condition [e.g., chronic kidney disease (CKD) and osteoarthritis (OA)] (*n* = 5, 14.7%). Other studies included patients with Amyotrophic Lateral Sclerosis (ALS; *n* = 1, 2.9%), patients with an unspecified terminal illness (*n* = 1, 2.9%), long-term care patients (*n* = 2, 5.9%), and patients with intracerebral hemorrhage (*n* = 1, 2.9%). Ten studies (25.6%) did not include a clinical sample. Most of the studies focused on patient decisions (*n* = 22, 64.7%), and the rest focused on a combination of patients/surrogates (e.g., caregivers and family members; *n* = 11, 32.4%) and healthcare professionals and patients (*n* = 1, 2.9%).

## 4 Discussion

Enhancing EoL care necessitates a nuanced approach that integrates assessments of DM ability alongside care and treatment preferences. Standardized measures capable of evaluating DM in patients with CI, dementia, and other conditions that may affect DM ability are crucial for both practitioners and for the advancement of research on this topic. EoL DM measures should ensure patients’ autonomy and enable healthcare providers, family members, and/or proxies to better respect and uphold patients’ wishes during the decision-making process. This study set out to review the range of existing measures of EoL DM, describe their characteristics, and identify gaps in the literature where further measurement strategies may be necessary. The results will both inform the state of the field of DM abilities in an EoL context and support the development of the ARMCADA suite of measures to assess multiple domains of DM in older adulthood.

Results from a multi-domain scoping review of DM in participants aged 45 + years, published between 2018 and 2023, found that only 34 articles included measures of EoL decision-making in older adults. Of those 34, there was little consistency in the use of standardized measures to assess end-of-life decision-making ability. The most commonly used measure was the MacCAT-T (24), followed by the Decisional Conflict Scale (25); however, the majority of articles employed a measure that was not used in any of the other studies in the dataset. As such, these findings suggest a critical gap in the current state of DM research: the lack of robust measures to assess DM ability in terminally ill patients, especially those with CI or dementia. Most existing measures tended to focus on decisional preferences and decisional outcomes rather than the ability to understand and make informed choices about EoL care options. Despite this, the inclusion of these measures was deliberate and grounded in the recognition that EoL decision-making is inherently multifaceted, influenced not only by cognitive ability but also by individual values, preferences, and perceived outcomes.

Understanding individuals’ preferences and perceived outcomes is essential not only for explaining their decision-making but also for guiding decisions when their ability to decide is diminished. In such cases, tools that incorporate these elements can help surrogates and clinicians infer and honor the individual’s values and wishes (28). Incorporating measures of preferences and outcomes alongside those of decision-making ability allows for an integrated understanding of EoL decision-making and lays the groundwork for the development of more robust and comprehensive assessment tools—ones that reflect the complexity of real-world decision-making and support more person-centered care. Future measurement efforts would benefit from building on this integrative approach, examining how these constructs interact, and exploring how such integration can improve the utility of DM ability assessments in clinical settings.

Our results also showed that very few measures identified in our search had been used or validated in populations with CI. Persons with CI and various dementias generally have substandard EoL care, including more severe untreated symptoms, increased suffering, and a generally lower quality of life compared to patients with cancer (6). Although CI patients may have a lower capability to express suffering and preferences at the end of their lives, it is important to assess this ability to establish an EoL plan in which decisions are not solely left in the hands of proxies, healthcare providers, and/or family members. Assessing EoL-focused DM ability at the onset of symptoms and as the disease progresses is one way in which persons with CI and dementia can take an active role in their EoL care and is the reason our search included such a broad age range.

While cognitive impairment and clinical status is an important factor influencing EoL decision-making, it is equally important to acknowledge how cultural backgrounds, family dynamics, and spiritual beliefs play a profound role in the EoL decision-making process. For example, Rego et al. (9) found that spiritual wellbeing significantly correlated with better quality of life in palliative care and was associated with less decisional conflict. Furthermore, cultural norms may heavily influence whether decisions are to be made by an individual or their family members. In collectivist cultures, individuals tend to opt in for a shared decision-making process (29). While this scoping review did not primarily focus on such cultural dynamics, their relevance to EoL care underscores the need for a more holistic approach to evaluating and supporting decision-making, and future research should emphasize these additional dimensions.

Furthermore, the context of where EoL decisions happen can vary greatly and occur beyond the walls of hospital settings. Many of the measures identified in this scoping review relied on the presence of a physician or trained clinician capable of administering measures in a semi-structured interview format. Yet, this does not reflect the reality of many individuals nearing the end of their lives. In practice, EoL decisions are made in diverse environments, including hospices, long-term care facilities, and—increasingly—people’s homes. In such cases, physicians may not be present to assess one’s decision-making ability and preferences, despite how critical and time-sensitive these matters are. Given this variability in setting and varying levels of experience, tools that require extensive training, time, and in-person administration may not be practical. As such, the development of remote, culturally adaptable, brief, and easily administered assessments is essential. These tools must be both clinically valid and sensitive to the contextual nuances of EoL care, including cultural values, emotional dynamics, and cognitive and decision-making ability fluctuations that can occur rapidly as a person nears death. Development of such tools will ensure personal autonomy and that EoL decisions align with a person’s needs and wishes.

One limitation of the scoping review involves the specific search terms we used. For example, we included ‘advanced care planning’ and ‘terminal care’, which may not have fully captured individual decision-making and instead emphasized shared decision-making—a criterion excluded from this review. However, our use of inclusive terms such as “end of life” and “mental capacity” combined with our broad decision-making terms should still have ensured a comprehensive search. Another limitation is that our review excluded gray literature, and the results may be impacted by publication bias. Additionally, case studies, which were also excluded from the current review, may be able to offer further insights, particularly in scenarios where conventional approaches may not apply, such as middle-aged patients proactively undergoing decision-making ability testing in longitudinal studies spanning their lifespan until EoL. Many articles included in the current review did not provide much insight into the psychometric quality of the measures they used. Future research employing existing EoL decision-making measures, as well as studies developing new tools, should prioritize validating these instruments across diverse clinical populations, cultural contexts, and languages. This is crucial because the reliability and validity of a measure can vary significantly depending on the demographic and situational context in which it is applied. Ensuring cross-contextual robustness will enhance the generalizability and utility of these measures and therefore have the potential to greatly improve end-of-life care and decision-making processes of patients. Finally, to ensure relevance and support our goals in developing a novel battery of DM for the ARMCADA study, our search focused on papers published between 2018 and 2023, which may have led us to overlook foundational measures published before this time.

In conclusion, this scoping review highlights significant gaps in standardized assessments of end-of-life (EoL) decision-making (DM) abilities for older adults, particularly those with cognitive impairment (CI) and dementia. The review underscores the scarcity of consistent, robust measures to assess DM ability, with the majority of studies employing unique, non-recurring measures. These findings emphasize the need for culturally relevant, adaptable, and validated tools that not only respect patient autonomy but also empower individuals with CI to engage in EoL planning from the onset of symptoms. The review also highlights a need to clearly distinguish between decision-making ability, decision-making capacity, decision-making competence, and decision-making preference, as this can greatly influence where it is used and what contexts it is suitable for. Addressing these gaps could improve the quality of EoL care for cognitively impaired populations and ensure that patients’ preferences are upheld. These results provide valuable insights to inform future research and the development of the ARMCADA suite of DM measures.

## Data Availability

The original contributions presented in the study are included in the article/Supplementary material, further inquiries can be directed to the corresponding author.
