# Supplementary material for: Identification and synthesis of end-of-life decision-making measures: a scoping review
Source: Front Med (Lausanne). 2025 Jul 31;12:1540486. doi: 10.3389/fmed.2025.1540486 (PMC12350294; doi:10.3389/fmed.2025.1540486)
Supplement: Supplementary file 1 [file Data_Sheet_1.docx]

Supplementary Material

# Table S1. Inclusion and exclusion criteria for articles (Ho et al., 2024*)*.

|  | Inclusion criteria | Exclusion criteria |
| --- | --- | --- |
| Population | The assessment was conducted with at least one group of individuals over 45 years. | Adults ≤ 45 years old |
| Study Characteristics | The study mentions at least one assessment of a DM target domain. The domain of interest is an outcome assessed by the study. | Single-subject research  Case studies  Focus group  Review articles  Narrative reviews  Grey literature including theses, conference papers, and preprints  Books and/or book chapters  Commentaries  Other non-research publications |
| Other | Language: All languages as long as manuscript is in English.  Location: All geographical locations. | Articles that only measure shared decision-making  Articles that only measure decision aids  Perceptual/Low-level decision making |

# Table S2. Measures used to assess end-of-life decision-making.

| **Measure Name** | **# of Citing Articles** | **Citing Articles** | **Measure Description** | **Age Ranges covered** | **Clinical Groups** | **In person vs. Remote** | **Format** | **Examiner vs. Self-Administered** | **Reporter** |
| --- | --- | --- | --- | --- | --- | --- | --- | --- | --- |
| MacArthur Competence Assessment Tool for Treatment (MacCAT-T) | 7 | Kiriaev et al., 2018; Kolva et al., 2018; Kolva et al., 2020; Occhiogrosso et al,, 2020; Poth et al., 2023; Santos, 2022; Spataro & La Bella, 2021 | Tool uses an interview and vignette format to measure four domains of competence: understanding information relevant to the condition and recommended treatment, reasoning of potential risks and benefits of choices regarding treatment, appreciation of the nature of the situation and potential consequences of the choice, and expression of a choice. Made specifically for treatment and medical decisions. | 45-85+ | - Nursing home residents - Terminally ill patients with cancer - Mild to Moderate Alzheimer's Disease (AD) - Dementia - Amyotrophic Lateral Sclerosis (ALS) - Patients w/ Malignant Gliomas - Terminally Ill Patients with Advanced Cancer | In person | Semi-structured interview  (w/ vignette) | Examiner | - Patient - Patient and caregiver - Patient and next-of-kin |
| Decisional Conflict Scale (DCS) | 5 | Huang et al., 2020; Rego et al., 2020; Song et al., 2019; Tan et al., 2019; You, 2019 | Tool designed to gauge the amount of uncertainty or difficulty a person experiences when making a decision. While it is commonly used in healthcare to evaluate how confident patients feel about their treatment choices, it can also be utilized in various other contexts. | 18-95 | - Palliative care outpatients (cancer); Dementia; - Advanced stage chronic illness; - End-stage renal disease | Hybrid | Questionnaire or Semi-structured interview | Self or Examiner | - Patient - Patient and family member - Patient and family caregiver |
| Decision Regret Scale (DRS) | 2 | Tan et al., 2019; Wilding, et al., 2020 | Tool designed to measure the extent of regret a person experiences after making a decision. It assesses how much an individual wishes they had made a different choice and their level of remorse or dissatisfaction with the outcome of the decision. | 45-85+ | - Renal disease (dialysis patients) - Prostate cancer | Hybrid | Questionnaire | Self | - Patient |
| End-of-life Care Preference Questionnaire | 1 | Ho et al., 2022 | Questionnaire with 5 domains: care preferences, life-sustaining treatment preferences, information preferences, decision-making preferences, and death issues. The DM domain contains four items: eagerness to be involved in DM for treatment plans, preferred decision-maker when patient is incapable, preferred person to discuss EoL issues with, and use of advance care planning. | 65-85+ | - None | In person | Questionnaire or Semi-structured interview | Self and Examiner | - Patient |
| End-of-life care survey | 1 | Waller et al., 2018 | Survey includes questions regarding preferred type of EoL care (extending life as long as possible, relieving pain as much as possible, or unsure), preferred location of EoL care, involvement in EoL decision-making preferences, and completion of advance care instruments. | 45-85+ | - Unspecified cancer | Remote | Questionnaire | Self | - Patient and support person |
| Problem-Solving Decision-Making scale (Portuguese version) | 1 | Gregório et al., 2020 | Scale presents 3 short vignettes: the morbidity vignette, the quality-of-life vignette, and the mortality vignette. Each vignette presents 6 tasks posing questions as to who should be responsible for treatment decisions, determining comfort, diagnosing the problem, etc. | 20-96 | - None | In person | Semi-structured interview (w/ vignettes) | Examiner | - Patient |
| Advance Care Planning – Capacity Assessment Vignettes Method (ACP–CAV) | 1 | Kiriaev et al., 2018 | Two vignettes present two medical situations (1) resuscitation which has a low probability of being successful and allowing a person to return to previous health (2) stomach cancer diagnosis in which a patient is given two options--surgery which results in a feeding tube or care that would focus on pain relief and good nursing care. Participants are then asked to explain the scenario, treatment options, choice, reason for choice, consequences of choice, and repeated expression of choice. | 60-85+ | - Nursing home residents | In person | Semi-structured interview (w/ vignettes) | Examiner | - Patient and next-of-kin |
| Advance Care Planning Engagement Survey- Traditional Chinese (ACP-TC) | 1 | Wei et al., 2022 | The survey evaluates future medical care decision-making processes in individuals regardless of age and health status. The long-form survey contains 82 items while the shortest version at present contains 2 subscales (4 questions) regarding: quality of life and medical decision-makers. | 18-84 | - None (some participants take long-term medication) | In person | Questionnaire | Not reported | - Patient |
| Advance Care Planning Questionnaire | 1 | Lim et al., 2022 | Questionnaire that contains domains associated with advance care planning including knowledge, attitude, justification (for or against ACP), and practice. | 22-88 | - None | In person | Questionnaire  Semi-structured interview | Examiner | - Patient and/or accompanying person |
| Advanced Care Planning (ACP) (interview/questionnaire) | 1 | Jin et al., 2022 | ACP interview and questionnaire that asks questions regarding preferences for ACP, concerns for ACP, and death preferences. | 36-98 | - Nursing home residents | In person | Questionnaire or Semi-structured interview | Examiner | - Patient and healthcare professionals |
| AIP-LTC decision-making | 1 | Lindquist et al., 2022 | Aging in place/ long term care needs assessed in an online survey format; questions include whether someone has plans if they have AD and are no longer able to live independently. | 65-84 | - None | Not reported | Questionnaire | Examiner | - Patient |
| Assessment of Capacity to Consent to Treatment (ACCT) | 1 | Kotzé et al., 2021 | A vignette is presented in which an individual has a stroke and there is a subsequent question asked about resuscitation if the person goes into cardiac arrest; the assessment focuses on impact of choices on valued activities/ relationships, considers individuals’ preferred DM style, and view on quality vs. length of life. | 60-84 | - Unspecified mental health illnesses | In person | Semi-structured interview (w/ vignette) | Examiner | - Patient |
| Confidence in surrogate's ability to make healthcare decisions consistent with the older adult’s wishes | 1 | Bravo et al., 2018 | Questions about satisfaction and confidence a surrogate can decide on behalf of an older adult consistent with the latter’s wishes. The question is asked at the interview's beginning, repeated at the end, and revisited 6 months later. | 65-85+ | - None | In person | Semi-structured interview | Examiner | - Patient and surrogate |
| Decisional balance, attitudes, practice behaviors of ACP (DAP-ACP) | 1 | Chen et al., 2022 | Thirty-four item self-report tool encompassing reporter’s decisional balance (individual’s assessment of the pros and cons in changing their decisions), values/ beliefs, and behavioral practices of ACP. | 18-85+ | - Advanced cancer patients | In person | Questionnaire | Self | - Patient |
| EoL-care decision scale | 1 | Huang et al., 2022 | Scale evaluates patient and family caregiver’s attitudes towards the use of life-sustaining treatment for a person with severe dementia: CPR, artificial respirator, and feeding tube on a 5-point Liker scale (0 being completely unacceptable to 5 being completely acceptable). | 45-85+ | - Cognitive Impairment (CI) | In person | Questionnaire | Examiner | - Patient and surrogate (family-caregivers) |
| Life-Support Preferences Questionnaire (LSPQ) | 1 | Ke et al., 2020 | Nine health-related scenarios (i.e. severe dementia, coma status with slight chance of recovery, terminal cancer with pain, etc.) are presented to individuals to help identify their preferences for EoL care. Each scenario includes 4 potential medical treatments and participants rate each treatment option for each scenario on a 5-point Likert scale (absolutely unwanted to absolutely wanted). | 65-85+ | - Some type of medical condition | In person | Questionnaire or Semi-structured interview | Examiner | - Patient and surrogate (family member or friend) |
| MacArthur Competency Assessment Tool for Clinical Research (MacCAT-CR) | 1 | Goswami et al., 2020 | The tool employs an interview and vignette format to evaluate four key aspects of competence: understanding relevant information about the condition and proposed treatment, reasoning through the potential risks and benefits of treatment options, appreciating the nature of the situation and the possible consequences of decisions, and expressing a choice. It is specifically designed to assess competence for agreeing to participate in clinical research. | 25-91 | - Advanced cancer | In person | Semi-structured interview (w/ vignette) | Examiner | - Patient |
| Patient Expectation for Participation in Medical Decision‐making Scale (PEPMDS) | 1 | Xiao et al., 2021 | Twelve-item self-report scale that assesses knowledge/information, and decisional preferences, and control. Items are ranked on a 5-point Likert scale. | 18-77 | - Colorectal, breast, or lung cancer | In person | Questionnaire or Semi-structured interview | Examiner | - Patient |
| Patients’ perspectives on dialysis treatment decisions and EoL Care (EoLC) survey (modified for current study) | 1 | Saeed et al., 2019 | Questionnaire asking patients about their knowledge of their condition, progression of their condition, palliative care services, and their perspectives on their prognosis, EoL care, and quality of life. | 45-84 | - Chronic kidney disease | Hybrid | Questionnaire | Self | - Patient |
| Sure of myself, Understand information, Risk/ benefit ratio and Encouragement (SURE) Test | 1 | Nair & Kohen, 2019 | Four-item assessment of decisional conflict asking participant if they are sure of their choice, whether they know the benefits and risks of their choice, whether it is clear to them what benefits and risks matter to the participant most, and whether they have enough knowledge and support to make a choice. | 45-84 | - Intensive Care Unit (ICU), telemetry, medical, or surgical ward patients | In person | Questionnaire or Semi-structured Interview | Examiner | - Patient |
| The ACP Acceptance Questionnaire | 1 | Yang et al., 2021 | Nineteen-item Likert scale focusing on attitudes towards ACP, feelings about ACP, and intentions towards ACP. |  | - Patients with at least one chronic condition | In person | Questionnaire | Self | - Patient |
| The Elderly Death Attitude Scale | 1 | Yang et al., 2021 | Twenty-five item 7-point Likert scale that assesses fear and anxiety of death, escape-oriented death acceptance, natural acceptance, approach-oriented death acceptance, and death escape. | 65-85+ | - Patients with at least one chronic condition | In person | Questionnaire | Self | - Patient |
| The Standard Gamble (SG) assessment | 1 | Slaughter et al., 2019 | Assessment consists of a series of choices made between a current health status and a hypothetical treatment that may either produce perfect health or a painless death at varying probabilities. | 49-80 | - Primary intracerebral hemorrhage | In person | Semi-structured interview (w/ vignette) | Examiner | - Patient and caregiver |
| The View of Relocation Scale (VRS) | 1 | Davison et al., 2022 | Twelve-item self-report measure designed to be completed after relocation to long-term care assessing perceived need and perceived control. | 62-99 | - Permanent long-term care (LTC) residents | In person | Questionnaireor Semi-structured interview | Examiner | - Patient |
| Underspecified questions about organ donation for a relative | 1 | Liu et al., 2021 | Questionnaire included questions about whether one would want to have their organs donated after death and whether they would donate their relatives' organs following their death. Overconfidence regarding their decisions was measured using a 5-point Likert scale. | 21-65+ | - None | Hybrid | Questionnaire | Not reported | - N/A |
| Healthcare related values/ End-of-life values and preferences | 1 | Kotzé et al., 2021 | Participants are asked to choose three valued activities from a list including being able to take care of oneself (activities of daily living), move around, live at home, think clearly, make own life decisions, have relationships with family and friends, practice religion/spiritual life, live without significant pain/ discomfort, or do specific activities/ hobbies. | 60-84 | - Unspecified mental health illnesses | In person | Semi-structured interview | Examiner | - Patient |
| Willingness to Accept Life-sustaining Treatment (WALT) | 1 | Batteux et al., 2020 | Three vignettes present life-threatening situations in which the probability that treatment will work ranges from 10 to 90 percent. Vignettes included a death scenario, a functional impairment scenario (bedbound), and a cognitive impairment scenario. Participants answered whether they would accept life-sustaining treatment for these scenarios for themselves and for their partner. | 59-81 | - None | In person | Semi-structured interview (w/ vignettes) | Examiner | - Patient and surrogate (their partner) |
| Goals of Care | 1 | Song et al., 2019 | Two vignettes are presented in which the responses include the following goals of care: delaying death as long as possible, focusing on treatment options that emphasize comfort and peace, or uncertainty. | 55-85+ | - Dementia | Hybrid | Semi-structured interview (w/ vignette) | Examiner | - Patient and family caregiver |
